# Protective Effect of Borage Seed Oil and Gamma Linolenic Acid on DNA: *In Vivo* and *In Vitro* Studies

**DOI:** 10.1371/journal.pone.0056986

**Published:** 2013-02-27

**Authors:** Inmaculada Tasset-Cuevas, Zahira Fernández-Bedmar, María Dolores Lozano-Baena, Juan Campos-Sánchez, Antonio de Haro-Bailón, Andrés Muñoz-Serrano, Ángeles Alonso-Moraga

**Affiliations:** 1 Departamento de Bioquímica y Biología Molecular, Facultad de Medicina, Instituto Maimónides de Investigaciones Biomédicas de Córdoba (IMIBIC/Universidad de Córdoba), Córdoba, España; 2 Departamento de Genética, Facultad de Ciencias, Universidad de Córdoba, Córdoba, España; 3 Instituto de Agricultura Sostenible, Consejo Superior de Investigaciones Científicas, Córdoba, España; IIT Research Institute, United States of America

## Abstract

Borage (*Borago officinalis* L.) seed oil has been used as a treatment for various degenerative diseases. Many useful properties of this oil are attributed to its high gamma linolenic acid content (GLA, 18:3 ω-6). The purpose of this study was to demonstrate the safety and suitability of the use of borage seed oil, along with one of its active components, GLA, with respect to DNA integrity, and to establish possible *in vivo* toxic and *in vitro* cytotoxic effects. In order to measure these properties, five types of assays were carried out: toxicity, genotoxicity, antigenotoxicity, cytotoxicity (using the promyelocytic leukaemia HL60 cell line), and life span (*in vivo* analysis using the *Drosophila* model). Results showed that i) Borage seed oil is not toxic to D. *melanogaster* at physiological concentrations below 125 µl/ml and the studies on GLA indicated non-toxicity at the lowest concentration analyzed ii) Borage seed oil and GLA are DNA safe (non-genotoxic) and antimutagenic compared to hydrogen peroxide, thereby confirming its antioxidant capacity; iii) Borage seed oil and GLA exhibited cytotoxic activity in low doses (IC_50_ of 1 µl/ml and 0.087 mM, respectively) iv) Low doses of borage seed oil (0.19%) increased the health span of *D. melanogaster*; and v) GLA significantly decreased the life span of *D. melanogaster*.

Based on the antimutagenic and cytotoxic effects along with the ability to increase the health span, we propose supplementation with borage seed oil rather than GLA, because it protects DNA by modulating oxidative genetic damage in *D. melanogaster*, increases the health span and exerts cytotoxic activity towards promyelocytic HL60 cells.

## Introduction

Many recently developed remedial therapeutic and preventive medicines include the use of traditional plant-based preparations. Borage (*Borago officinalis* L.) is an annual plant used from ancient times for culinary and medicinal purposes. Recently, interest in borage has been renewed because its seeds are considered as one of the best sources of gamma-linolenic (all *cis*-6,9,12-octadecatrienoic acid (GLA)). This unusual fatty acid is an intermediate of indispensable compounds in the body such as prostaglandin E1 and its derivatives [1]. Borage seed oil has been promoted as an effective treatment for different pathologies, such as acute respiratory distress syndrome [2], [3], rheumatoid arthritis [4], atopic dermatitis, diabetic neuropathy and menopause-related symptoms [5]. It has also been shown to decrease inflammation, improve bone health [6] and exhibit beneficial effects on the function of the skin [7] and on the regulation of lipid metabolism [8].

Many properties of the borage seed oil are attributed to the GLA content, which constitutes 15–22% of the oil [9]. GLA is obtained from very few vegetable oils, including borage seed oil. This n-6 polyunsaturated fatty acid is used for prevention and/or treatment of various degenerative pathologies such as osteoporosis [10], diabetes [11] and cancer [12]–[14]. Additionally, it has been shown to suppress *in vitro* tumour growth [15], [16], improve oxygenation status [17], exert anti-inflammatory activity and display beneficial effects in the early stages of sepsis [18], [19].

The selected model for the study of the *in vivo* biological effects of borage seed oil and GLA is widely used for screening of substances with potential beneficial/harmful effects on humans. *Drosophila* has been a powerful genetic model system used in many fields of biology, as a model for human neurodegenerative diseases [20] reproductive toxicity [21] and others [22]. Today it is considered an excellent alternative animal model because the *Drosophila* genome annotation revealed that 70% of human genes have orthologs in *Drosophila* [23] and a 65–70% of functional homology with humans is documented [24].

The purpose of this study was to demonstrate the safety and suitability in the use of borage seed oil along with one of its active components (GLA) with respect to DNA integrity and to establish possible *in vivo* geno/antigenotoxic and *in vitro* cytotoxic effects. In order to measure these properties, five types of assays were carried out: genotoxicity, antigenotoxicity, toxicity and life span (*in vivo* analysis using the *Drosophila* model) and cytotoxicity (using the promyelocytic leukaemia HL60 cell line).

## Materials and Methods

### Borage seed oil and GLA

Commercial borage seed oil obtained by first cold pressure of seeds from organic cultivation was provided by Biolasi (Cat. N°. 40.03.500/SS, Biolasi, Spain). Organic farming methods for borage seed production in combination with cold-pressing methods allow the maximum content and recovery of antioxidants in the seed oil.

Gamma linolenic acid (all *cis*-6,9,12-octadecatrienoic acid), grade analytical standard (>99,0%) was purchased from Sigma (Cat. N°. L2378, Sigma-Aldrich, St. Louis, MO),

### Fatty acid analysis

Fatty acid composition of borage seed oil was determined by gas chromatography coupled with flame ionization detector (GC-FID). Fatty acid methyl esters of the borage seed oil were prepared according to Garcés and Mancha [25] and analyzed on a 7890A model gas-liquid cromatograph (Agilent Technologies, Santa Clara CA, USA). The capillary column used was RESTEX 2330 (60 m×0.25 mm i.d., 0.20 µm (Restek Corp, Bellefonte, PA, USA) containing 90% biscyanopropyl and 10% phenylcyanopropyl polysiloxane. The initial column temperature was 175°C (held for 19 min), then increased to 200°C at a rate or 5°C/min, and held for 14 min at 200°C. Fatty acids were identified by comparing the retention times of the fatty acids of the borage seed oil with those of known mixtures of standard fatty acids purchased from Sigma-Aldrich (USA) run on the same column under the same conditions.

### Triglyceride analysis

The triglycerides of the borage seed oil were separated by HPLC according to the COI T.20 Doc 25 method [26]. The isocratic elution was monitored by a refractive index detector. The mobile phase was acetone/MeCN 60:40 (v/v) at a flow rate of 0.6 ml/min at 15°C. All solvents were HPLC-grade.

### Tocopherol analysis

The tocopherol isomers in borage seed oil were determined in accordance with the IUPAC method 2432 [27] by using HPLC (Agilent 1100 series, Palo Alto, CA), with a G1314A detector and a 250 mm×4 mm, 5 µm, Spherisorb SS NH_2_ column). Results were adjusted for the average specific gravity of borage seed oil and expressed in mg/kg.

### *Drosophila* strains

Analysis of the genotoxic and antigenotoxic activities of borage seed oil and GLA, was evaluated by the *Drosophila* SMART test [28]. Two different *Drosophila melanogaster* strains carrying visible wing genetic markers were used: the flare (*flr*) *s*train *flr^3^/ln (3LR) TM3, Bd^s^* and the multiple wing-hair (*mwh*) strain *mwh/mwh*. The marker multiple wing hairs (*mwh, 3_0.3*) is a recessive mutation that is viable in homozygous flies, producing multiple hairs per cell instead of the wild type single-hair trichome [29]. The marker flare (*flr^3^, 3_38.3*) is a recessive mutation which produces individual wing hairs that are malformed. The *flr^3^* allele is retained via a balancer chromosome carrying multiple inversions and a dominant marker which is homozygous lethal (*TM3, Bd^S^*: Third Multiple 3, Beaded-Serrate) [30]. Additional information on genetic markers can be found in [31].

### Toxicity, Genotoxicity and Antigenotoxicity Procedures

Crosses were performed with optimally fertile virgin females of the *flr3/TM3*, *Bd^s^* strain and *mwh/mwh* males. After 8 h egg-laying, 72±3 h old larvae were washed in distilled water, transferred to fresh vials with 0.85 g *Drosophila* Instant Medium (formula 4–24, Carolina Biological Supply, Burlington NC, USA) which were wetted with 4 ml after of put solutions of increasing concentrations of the borage seed oil and GLA. Test groups consisting of 100 larvae each were: (i) negative control (distilled water); (ii) mutagenic positive control (hydrogen peroxide 0.12 M); (iii) 5 vials with increasing concentrations of borage seed oil (12.5, 18.7, 31.2, 62.5 and 125 µl/ml); and (iv) 5 vials with increasing concentrations of GLA (8.9, 13, 22, 45 and 90 mM). Taking into account the average daily food intake of *D. melanogaster* (1 mg/day) and the average body weight of *D. melanogaster* individuals (1 mg), the concentration range for both borage seed oil and GLA were calculate to fit them into the recommended fat daily intake for humans (60 g of total fat/day for 60 kg human body weight). Emerging adults of all groups were counted and stored in 70% ethanol to be analysed. Procedures are: 1) Toxicity assays evaluated (number of individuals born in treatment/number of individuals born in the negative control)x100. 2) Genotoxicity assays evaluated mutation rate diagnosis of different groups and 3) Antigenotoxicity testing, combined treatments were performed as previously described by Anter et al., 2011 [32], by using hydrogen peroxide 0.12 M as the genotoxicant and the concentrations of borage seed oil and GLA mentioned above in groups (iii) and (iv), evaluating mutation rate diagnosis of different treatments.

Marker-heterozygous genotypes (*mwh/flr^3^*) were mounted on slides with Faurés solution. Both dorsal and ventral surfaces of the wings containing 22,000 cells were screened under a photonic microscope at 400× magnification for the occurrence of individual spots (mwh or flr phenotype) or twin spots (mwh clone adjacent to flr clone). Small individual spots with one or two cells exhibiting the mwh phenotype corresponded to gene mutation and somatic recombination between the two marker genes occurring during the last mitotic rounds in the imaginal discs of the larvae. Large individual spots with three or more cells showing mwh or flr phenotypes corresponded to mutational events occurring earlier during larvae development. Twin spots with two juxtaposed clones corresponded uniquely to recombination events between the *flr^3^* gene and the centromere.

### Longevity assays

Animals undergoing longevity experiments exhibited the same genotype as in genotoxicity assays in order obtain comparable results. The F1 progeny from *mwh* and *flr^3^* parental strains produced by 24 h egg-laying in yeast was used. Longevity experiments were carried out at 25°C, following the procedure described by Chavous et al., 2001 [33]. Briefly, synchronised transheterozygous 72±12 h old larvae were washed in distilled water and separated into groups of 100 individuals in vials with a mixture of 0.85 g *Drosophila* Instant Medium and 4 ml of the different concentrations of the two substances selected. Emerging adults were collected, anesthetized under CO_2_ and placed in 1 ml longevity vials in groups of 10 individuals. Three replicates were used during the complete life span for each control and concentration established. The number of survivors was counted and the medium renewed twice a week.

### Cell cultures

The human promyelocytic leukaemia cell line HL60 was obtained from Dr. José M. Villalba Montoro (Department of Cell Biology, Univ. Cordoba, Spain) [32], [34]. HL60 cells were incubated in RPMI-1640 medium (Cat N° A1049101, Invitrogen) supplemented with: L-glutamine 200 mM, (Cat N° G7513, Sigma-Aldrich, St. Louis, MO) antibiotics 100× (Cat N° A5955, Sigma-Aldrich, St. Louis, MO) and 10% heat-inactivated foetal bovine serum (Cat N° SO1805, Linus). Cells were maintained at 37°C in an atmosphere of 5% CO_2_. Cultures were plated at a density of 12.5×10^4^cells/ml in 40 ml culture bottles (25 cm^2^) and passed every 2 days.

### Cytotoxicity assays

For the assessment of cell viability, HL60 cells were placed in 12 well culture plates (1×10^5^ cells/ml) and treated for 72 h with increasing concentrations of borage seed oil and GLA. Cell viability was determined by the Trypan Blue dye exclusion test (T8154, Sigma-Aldrich, St. Louis, MO). Cells were counted by adding an aliquot of 10 µl of the culture to 10 µl of the Trypan Blue dye. The mix was counted under a light inverted microscope using a Neubauer chamber (AE30/31, Motic). Aliquots were taken after 24, 36, 48, 60 and 72 h incubation. After each incubation period, a growth curve was established and IC_50_ values (concentration of tested compound causing 50% inhibition of cell growth) were estimated. Curves are expressed as the percentage of survival with respect to controls at 72 h of growth.

### Statistical analysis

For the evaluation of genotoxic effects, the frequencies of spots per fly of each treated series were compared to the concurrent negative control for each class of mutational clone. A multiple-decision procedure was used to categorize results as positive, weakly positive, inconclusive, or negative [35]. Statistical analyses were carried out for single, large, twin and total number of spots recovered. Inconclusive and positive data were evaluated by the non-parametric *U* test of Mann, Whitney and Wilcoxon according to Frei and Würgler 1995 [36]. The inhibition percentage for each compound and treatment was calculated following Abraham, 1994 [37] (hydrogen peroxide alone - substance assayed + hydrogen peroxide / hydrogen peroxide alone) × 100.

In addition, the Kaplan-Meier estimates of the survival function for each concentration and respective control are plotted as survival curves. Statistical analyses and significance of the curves were assessed using the SPSS 15.0 statistics software (SPSS Inc. Headquarters, Chicago, IL, USA) using the Log-Rank (Mantel-Cox) method.

Viability curves of leukaemia cells were plotted as mean viability ± standard error of three independent replicas for each compound and concentration.

## Results

### Borage seed oil analysis

The data given in Table 1 show the fatty acid, triglyceride and tocopherol composition of borage seed oil.

**Table 1:** Fatty acids, triglyceride and tocopherol composition of borage seed oil.

Fatty acid composition			
Fatty acid	%	Fatty acid	%
Palmitic acid (C16:0)	10.7	γ -Linolenic acid (C18:3)	21.1
Stearic acid (C18:0)	6.4	Eicosenoi c acid (C20:1)	4.2
Oleic acid (C18:1)	18.5	Erucic acid (C22:1)	2.3
Linoleic acid (C18:2)	36.6	Others	0.2

Triglyceride composition (1)			
Triglyceride	%	Triglyceride	%
LnLnLn	0.28	SLLn	3.30
LLnLn	5.24	POLn	6.47
LLLn	8.96	OOL	5.60
OLnLn	1.06	ELL	1.07
LLL	6.03	OLnE	2.76
OLLn	8.78	PLO	8.03
PLLn+SLnLn	5.92	SOLn	1.78
OLL	10.90	PLP+OOO	3.89
OOLn	2.78	SOL	3.16
PLL	4.30	POO	2.29
Others	7.4		

Tocopherol composition			
Tocopherol	mg/kg	Tocopherol	mg/kg
γ-tocopherol	39	δ-tocopherol	1320

(1) (P = palmitic, S = stearic, O = oleic, L = linoleic, Ln = gamma-linolenic, E = eicosenoic)

Unsaturated oleic (*cis*-9 octadecenoic), linoleic (all *cis*-9,12-octadecadienoic) and gamma-linolenic acid (all *cis*-6,9,12-octadecatrienoic acid), were the major fatty acids of the oil, representing the 18,5%, 36,6% and 21,1% of the oil respectively. Borage seed oil contained a very high proportion of unsaturated fatty acids to saturated fatty acids (82,7% to 17,1%), and these data are in agreement with those reported in previous studies [9], [38]–[40].

Borage oil contains a complex triglyceride profile. More than 20 different triglycerides in the borage seed oil have been determined by high-performance liquid chromatography (Table 1). Dilinoeoyl-oleoyl-glycerol (OLL) was the major triglyceride, followed by dilinoleoyl-linolenoyl-glycerol (LLLn), oleoyl-linoleoyl-linolenoyl-glycerol (OLLn), palmitoyl-linoleoyl-oleoyl-glycerol (PLO) and palmitoyl-oleolyl-linolenoyl-glycerol (POLn) with values of 10.90, 8.96, 8.78, 8.03 and 6.47% respectively.

Only gamma tocopherol and delta tocopherol were present in the borage seed oil (Table 1). Delta tocopherol, with a concentration of 1320 mg/kg represents more than 97% of the total tocopherol content. These tocopherol profile and concentration are similar to that reported in a previous work [41]

### Toxicity analysis

Toxicity assays showed that borage seed oil is not toxic to *D. melanogaster* at concentrations below 125 µl/ml, presenting an average survival rate of 80% and no dose effect was observed. However, combined treatments of borage seed oil and hydroxide peroxide reduced the survival rate more than 50% (when compared with the treatment using borage seed oil alone). The concentrations 62.5 µl/ml to 125 µl/ml decreased survival considerably in a dose-dependent manner. This range of oil concentration in the medium (6,25 to 12,5%) is similar to that used with other vegetable oils in a previous work [42].

However, the data on GLA indicated non-toxicity at the lowest concentration resulting in a survival rate of 99%, although the next higher concentration tested showed a 66% decrease in survival and higher concentrations showed a strong dose-dependent effect.

In summary, taking the average toxicities into account (borage seed oil 79%, borage seed oil plus hydrogen peroxide as a toxicant 42%, GLA 55% and GLA plus hydrogen peroxide 31%) a general pattern of responses was observed indicating that treatments with borage seed oil and GLA, resulted in strongly reduced survival rates when combined with hydrogen peroxide (Table 2).

**Table 2:** Toxicity levels of borage seed oil and GLA in *D. melanogaster*.

Borage seed oil (µl/ml)	Survival %		Gamma-linolenic acid (mM)	Survival %	
	Simple treatment ^(1)^	Combined Treatment ^(2)^		Simple treatment ^(1)^	Combined Treatment ^(2)^
0	97.11	84.44^(4)^	0	97.11	84.44^(4)^
12.5	95.55	61.11***	8.9	99.25	48.88***
18.7	84.44	42.22***	13	66.66***	22.22***
31.2	90	53.33***	22	39.25***	39.25***
62.5	100	31.11***	45	50.37***	37.77***
125	28.88*** ^(3)^	24.44***	90	20.74***	32.59***

1 Data are expressed as percentage of survival adults with respect to 450 untreated 72 h old larvae from three independent experiments. ^2^Combined treatments using standard medium and 0.12 M hydrogen peroxide. ^3^Asterisks indicate significance levels with respect to the untreated control group: * p≤0.05; ** p≤0.01; ***p≤0.001. ^(4)^ Survival percentage for 0.12 M hydrogen peroxide alone treatments.

### Geno/antigenotoxic activity

Table 3 shows the results obtained from 72 h genotoxicity studies after simple treatments of transheterozygous larvae of *D. melanogaster*. The genotoxicant hydrogen peroxide resulted mutagenic as expected [27], doubling the mutation rate of the negative water control (and ensuring the adequacy of the assay). None of the compounds studied were genotoxic in the somatic mutation and recombination wing spot assay at any concentration tested. As no dose-effect was observed it is estimated that borage seed oil and GLA induced weighted averages of 0.31 an 0.23 spots/wing respectively, which were not significantly different from the negative water control (0.25 total clones/wing). The toxic effect observed for GLA do not correspond with genotoxic effect as the genotoxicity in the SMART assay is screened on the wing surface of the survival adults. The results of antigenotoxicity assays, shown in Table 4 indicated that borage seed oil and GLA were able to desmutagenise the genotoxic activity of hydrogen peroxide although no dose-effect was observed. The inhibitory potential against hydrogen peroxide of the two substances, borage seed oil and GLA were similar, yielding weighted averages of 38.2% for borage seed oil and 38.1% for its major constituent GLA.

**Table 3:** Genotoxicity of *borage* seed oil and gamma-linolenic acid in the Somatic Mutation and Recombination Test.

Genotoxicity analysis					
	Mutation rate (Spots per wing) diagnosis (1)				
	Small single spots 1–2 cells; m = 2	Large single spots > 2 cells; m = 5	Twin spots m = 5	Total spots m = 2	N° of emerging adults
*Controls*					
(N° of scored wings)					
H_2_O (297)	0.23 (69)	0.01 (4)	0.01 (3)	0.25 (75)	349
H_2_O_2_ 0.12 M (272	0.46 (126)	0.03 (12)	0.01 (3)	0.51 (141) (+)	328
*Borage seed oil* µl/ml (N° of scored wings)					
12.5 (41)	0.24 (10)	0.05 (2)	0	0.29 (12) (−)	52
18.75 (39)	0.28 (11)	0.1 (4) (+)	0	0.41 (16) (−)	53
31.25 (40)	0.3 (12)	0.05 (2)	0.03 (1)	0.37 (15) (−)	48
62.5 (24)	0.12 (3)	0	0.04 (1)	0.16 (4) (−)	26
125 (4)	0	0	0	0 (−)	9
*Gamma-Linolenic Acid* mM (N° of scored wings)					
8.9 (94)	0.21 (20)	0.021 (2)	0.01 (1)	0.24 (23) (−)	106
13 (40)	0.15 (6)	0	0	0.15 (6) (−)	49
22 (18)	0.22 (4)	0	0	0.22 (4) (−)	22
45 (33)	0.3 (10)	0.03 (1)	0	0.33 (11) (−)	30
90 (4)	0	0	0	0 (−)	7

(1) Statistical diagnoses according to Frei and Würgler (1988, 1995) [35], [36]: + (positive, genotoxic), - (negative, non genotoxic). Significance levels α = β = 0.05, one-sided test

**Table 4:** Antigenotoxicity of *borage* seed oil and gamma-linolenic acid in the Somatic Mutation and Recombination Test.

Antigenotoxicity analysis							
	Mutation rate (Spots per wing) diagnosis (1)						
	Small single spots (1–2 cells) m = 2		Large single spots (>2 cells) m = 5	Twin spots m = 5	Total spots m = 2	% of inhibition^(2)^	N° of emerging adults
*Controls*							
(N° of scored wings)							
H_2_O (297)		0.23 (69)	0.01 (4)	0.01 (3)	0.25 (75)		349
H_2_O_2_ 0.12 M (272)		0.46 (126)	0.03 (12)	0.01 (3)	0.51 (141) (+)		328
*Borage seed oil* µl/ml (N° of scored wings)							
12.5 (40)		0.27 (11)	0.075 (3)	0	0.35 (14) (−)	31	43
18.75 (24)		0.16 (4)	0	0	0.16 (4) (−)	68	27
31.25 (26)		0.23 (6)	0.07 (2)	0	0.3 (8) (−)	41	25
62.5 (24)		0.37 (9)	0	0	0.37 (9) (−)	27	28
125 (12)		0.41 (5)	0	0	0.41 (5) (−)	19	9
*Gamma linolenic acid* mM (N° of scored wings)							
8.9 (50)		0.3 (15)	0.02 (1)	0	0.32 (16) (−)	37	67
13 (12)		0.25 (3)	0	0	0.25 (3) (−)	51	15
22 (28)		0.39 (11)	0	0.03 (1)	0.42 (12) (−)	17	27
45 (26)		0.19 (5)	0.03 (1)	0.03 (1)	0.26 (7) (−)	49	29
90 (16)		0.18 (3)	0.06 (1)	0	0.25 (4) (−)	51	11

H_2_O_2_ 0.12 M used as a genotoxicant.

(1) Statistical diagnoses according to Frei and Würgler [35], [36]: + (positive, genotoxic), - (negative, non genotoxic). Significance levels α = β = 0.05, one-sided test. ^(2)^ Inhibition percentage calculated according to Abraham (1994) [37].

### Cytotoxic activity

**Figure 1:**
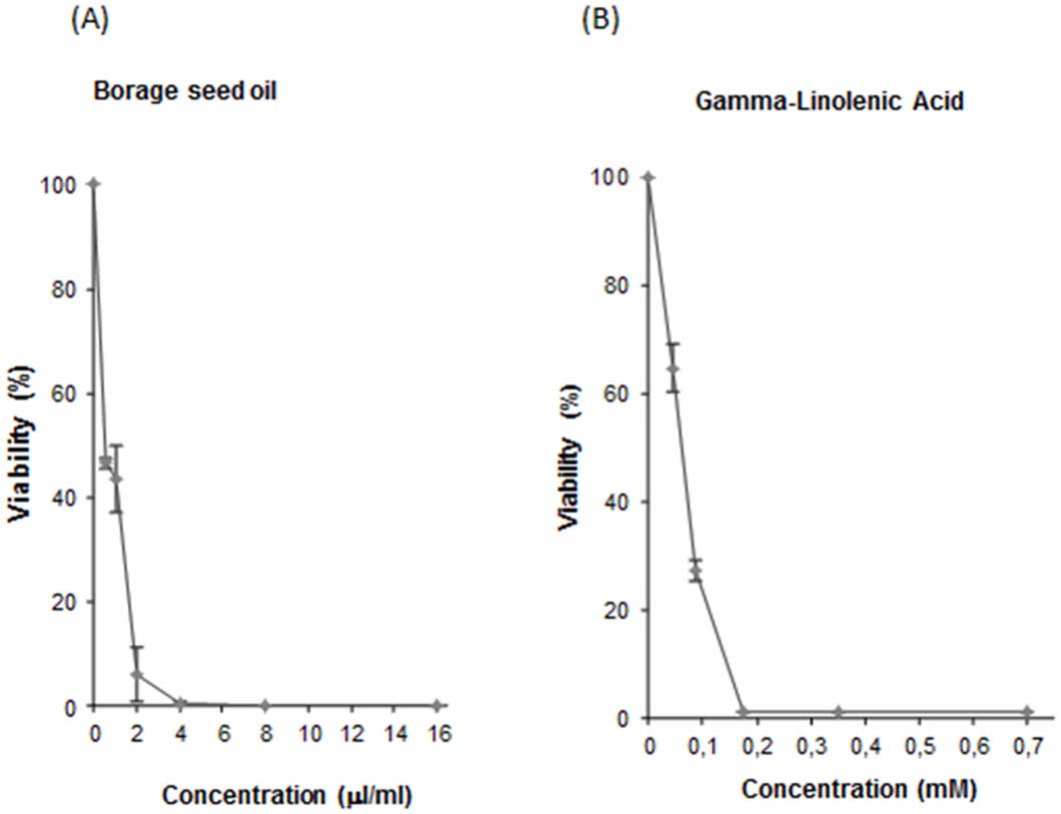
Viability of HL60 cells treated with borage seed oil (A) and GLA (B) for 72 h. Curves are plotted as percentages with respect to the control counting from three independent experiments (mean ± SD).

Borage seed oil and GLA showed to be cytotoxic for HL60 promyelocytic cells in a dose-dependent manner displaying inhibitory concentrations 50 (IC_50_) of 1 µl/ml and 0.087 mM, respectively. The concentrations of GLA used in the cytotoxicity experiments corresponded to the content of GLA of each oil dose. The cytotoxic effect of borage seed oil could almost entirely be due to the GLA content of these concentrations, deducible from the similarity of the curve shapes (Figure 1).

### Longevity assays

**Figure 2:**
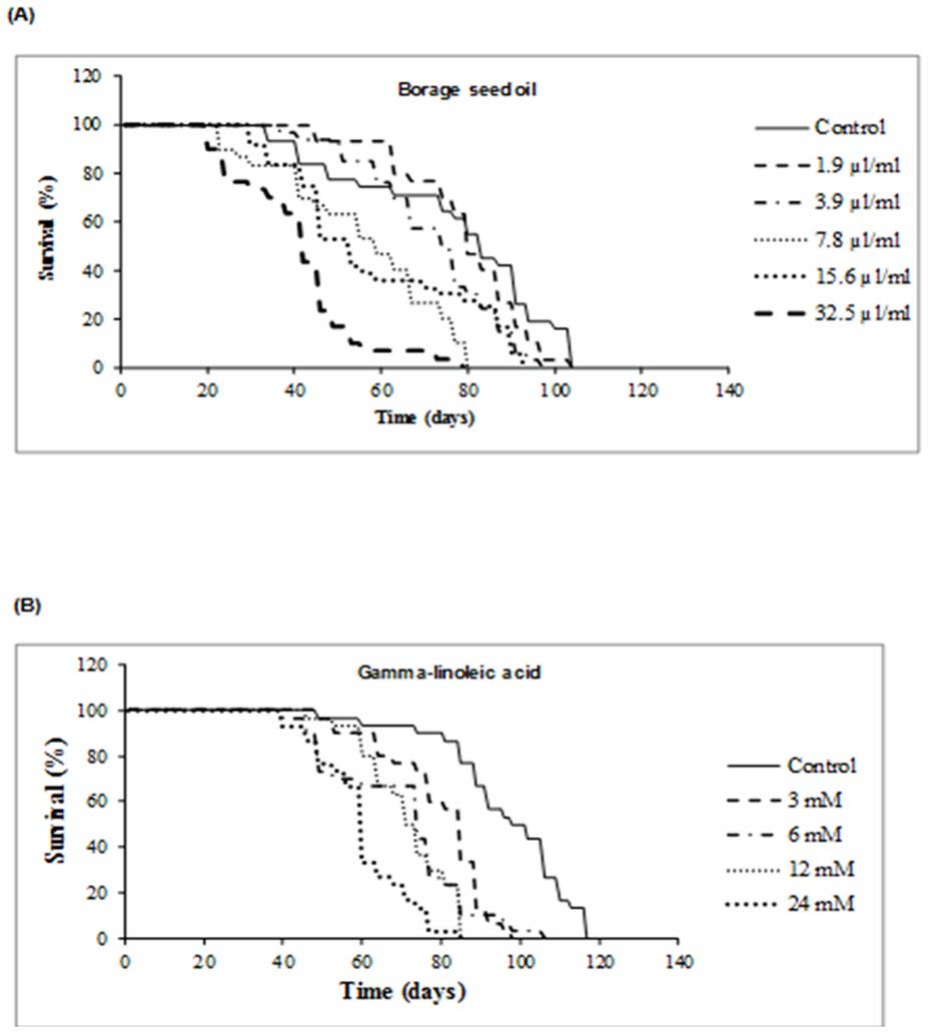
Effect of borage seed oil (A) and GLA (B) supplementation on the life span of *Drosophila melanogaster*.

When compared to acute toxicity treatments, lower concentrations were applied to larvae for longevity tests, as these treatments were chronic. Total lifespan curves are plotted in Figure 2 for borage seed oil (A) and GLA (B). Borage seed oil (0.19%) and control groups reached comparable maximum survival levels of 104 days, with mean values of 77 and 81 days, respectively. Although differences observed were not significant (p≤0.6), additional information related to the quality of life could be obtained from the peaks of these life span curves. Analysis of the 75^th^ percentile showed enhanced survival of the two lowest concentration borage seed oil groups (77 and 63 days, respectively), compared to the control group (55 days). We compared the survival curves ≥ 75%, of living flies using water as control with the remaining substances. This part of the life span is considered as the health span of a curve, characterized by low and more or less constant age-specific mortality rate values [43]. The mean survival of the control group exceeding 75% of the lifespan curves was 42 days, in contrast to 60 and 51 days survival of groups treated with 0.19% and 0.39% borage seed oil (p≤0.002 and p≤0.02, respectively). Increasing concentrations of borage seed oil showed significant but negative values compared to controls by decreasing the life and health span of the flies. By contrast, Figure 2 (B) shows that none of the assayed concentrations of GLA increased the life span of *D. melanogaster*, but decreased the mean survival time significantly.

## Discussion

Pleiotropic activities with beneficial effects have been attributed to borage seed oil for the treatment of several disorders such as acute respiratory distress syndrome, rheumatoid arthritis, diabetic neuropathy, menopause and atopic dermatitis [3]–[5]. Here, we present the first study showing results regarding the toxicity, genotoxicity, antigenotoxicity and longevity properties, of borage seed oil along with one of its active components (GLA). The study was performed *in vivo*, in the eukaryotic system *D. melanogaster* because more than half of its genes are known to have mammalian homologues [44], [45]. *D. melanogaster* provides an excellent *in vivo* genetic model system for toxicity studies [20]. *Drosophila* has been used to model human conditions such as neurodegenerative and infectious diseases [46], [47], immune system [48] cardiac function [49], and aging [50]. From a toxicological point of view *Drosophila* and higher mammals have similar dose–response relationship with many compounds such as monofunctional alkylating agents [51]. Thus, we believe that laboratory-based experimental evidences using this model is useful in generating information that could be of value for their efficient extrapolation to higher organisms.Therefore the information obtained regarding genotoxicity in an excellent indicator of the genetic safety of treatments with borage seed oil. Borage seed oil was toxic only at the highest assayed concentration (12,5%). This concentration equals to 7.5 g/day in humans subjects of 60 kg body weight. This fat intake represents 12,5% of the daily fat intake, which is under the Acceptable Macronutrient Distribution Range for fat (20–35%) of energy for total fat [52].This concentration is slightly higher than the usually recommended dosage (1–6 g/day) in chronic treatments for various degenerative pathologies [53], [54]. Thus, survival assays were performed with concentrations below 125 µl/ml.

More than 200 effects of hydrogen peroxide as a genotoxin have been previously described [55], and there are several antigenotoxic studies that demonstrate its genotoxic potency, being both mutagenic (oxidative genetic damage induced by the production hydroxyl radicals through the metal-catalyzed Fenton reaction) and recombinogenic (genotoxic mechanism involved as an early step in the origin of some types of human cancers) [34], [56]–[58]. Borage seed oil and GLA genotoxicity assays confirm the safety of these compounds which is the first step in characterizing their usefulness as a neutraceuticals. Besides this, our antigenotoxicity assays demonstrated for the first time that both borage seed oil and GLA exert a role in the genomic stability of *D. melanogaster*, acting as a desmutagenic agents against hydrogen peroxide by scavenging the reactive oxygen species originated by the model genotoxicant used. The similarity in the antigenotoxic inhibitory potencies of GLA (38.1%) and borage seed oil (38.2%) as well as the high content of GLA in borage seed oil (21.1%) indicates that GLA may account for the antigenotoxic ability of borage seed oil.

Additionally, our results show that both substances tested have a cytotoxic effect on HL-60 tumour cells. Specific mechanisms inducing cytotoxicity have been observed for GLA in other studies: i) GLA induces apoptosis in this cell line checked by DNA fragmentation hallmark [59]; ii) GLA enhances the cytotoxicity of docetaxel in human breast cancer cells by mechanisms other than lipoperoxidation, and the GLA-induced transcriptional repression of HER-2/neu oncogene might be one component of the mechanisms [60]; iii) human chronic myelogenous leukaemia K562 cells are also switched to the apoptotic way by activation of caspase-3 and release of cytochrome c [13]; iv) GLA treatment in hepatocellular carcinoma Hhh7 cell upregulated genes encoding antioxidant proteins [12]; and v) in the Lobund-Wistar animal model of prostate cancer by treating rats with NMU and supplementing with GLA a decrease of prostate growth was observed [15]. Additionally, it is known that intra-tumoural administration of GLA can induce regression of human gliomas [61] and GLA linked to lithium salt is able to inhibit cell growth of two pancreatic tumour cell lines (MIA PaCa2 and Panc 1) [62]. Therefore, our results are consistent with those of other authors, although they used different cell lines, and confirm the ability of cancer cell growth inhibition by GLA.

The cytotoxic effect of polyunsaturated fatty acids particularly arachidonic acid and the eicosanoids generated from it, is long known, being characterized as apoptotic and associated with oxidative stress. Chen et al., (1998) show that pretreatment with 0.03 mM arachidonic acid causes significant toxicity to the human hepatoma HepG2 cells (E9 cells) that over express human CYP2E1. The PUFA toxicity is associated with increased lipid peroxidation and can be diminished by antioxidants that prevent lipid peroxidation (such as ascorbic acid, Trolox and alpha tocopherol phosphate. Compared with arachidonic acid, oleic acid (C18:1) showed no significant toxicity to the E9 cells even at concentrations (0.05 mM) at which arachidonic acid is highly cytotoxic [63].

Similarly, Pompeia et al., (2002) demonstrated the cytotoxic effect of arachidonic acid in leukocytes (HL-60, Jurkat and Raji cells). Use this fatty acid was also demonstrated to have an apoptotic effect at low concentrations (10–400 µM) and a necrotic effect at high concentrations (400–1600 µM) [64].

Anti-ageing and anti-degenerative assays can be carried out using different *D. melanogaster* strains in order to perform life span trials with specific chronic diets in controlled environments [65], as the key interest for humans is to increase life span. In this work, we describe for the first time a study designed to examine the effects of both borage seed oil and GLA dietary supplementation on the life span of *D. melanogaster*. This animal model is an excellent system to investigate longevity-promoting properties of compounds and nutraceutical extracts. *Drosophila* has a short life span, can be cultured with simple diets, and represents a rich genetic resource with a fully sequenced genome. The life span of this insect is relatively short and adults seem to show many of the cell senescence features seen in mammals [65]. In this study, we demonstrate that low doses of borage seed oil are able to increase the health span portion ≥ 75% of the life span curves in *D. melanogaster* (Figure 2A). No positive effects on life/health span in Drosophila melanogaster have been found at any concentration of GLA (Figure 2B) The *Drosophila* strains used in this work is not extended-life mutants. Average life span data of *D. melanogaster* vary widely and are strongly dependent on rearing conditions. The average control life span (between 33 and 80 days) found in previous works [66]–[68], are lower than that found in the present study. Dietary supplementation does not always result in an increased life span. Dietary supplementation using different concentrations of substances such as nectarine, cocoa, broccoli or lamotrigine also resulted in values lower than with a normal diet (10% sugar and 10% yeast extract), dietary restriction (2.5% sugar and 2.5% yeast extract), or anoxia treatments [43], [68]–[70]. Our results show that borage seed oil did not reduce the life span, but increased significantly the health span portion of the life span curve.

The negative effects of GLA on the longevity trials of *D. melanogaster* are in agreement with the toxicity results for this fatty acid (Table 2) where GLA is shown to be more toxic than borage seed oil. Nevertheless, the genotoxic/antigenotoxic and cytotoxic assays with borage seed oil and GLA showed the same positive effect. This apparent mismatch could be due to the fact that GLA as free fatty acids is highly prone to the auto-oxidation process producing hydroperoxides and other oxygenated compounds that can reduce the average life span [71]. Besides this, GLA and other fatty acids contained in borage seed oil are part of a triacylglycerol molecules that is more stable and less susceptible to auto-oxidation [72], [73]. Tso et al. (2002) studied the intestinal absorption and lymphatic transport of two different seed oils containing GLA using a lymph fistula rat model, and demonstrated that the oleic, linoleic and gamma linoleic acid content of the dietary oils consumed are preserved in the fatty acid composition of lymph triglycerides [74].

Taking into account the whole toxicity, geno/antigenotoxicity, lifespan and cytotoxicity assays performed in the present study, the beneficial effects of borage seed oil described may be due not only to the presence of GLA but also to other major constituents, like oleic and linoleic acids, delta tocopherol and gamma-tocopherol (Table 1) with antioxidant and contrasted beneficial properties for various degenerative pathologies [12], [13], [75], [76].

Based on the findings of the present study, we conclude that i) borage seed oil is non- toxic to *D. melanogaster* at concentrations below 125 µl/ml and the studies on GLA indicated non-toxic at the lowest concentration used ii) borage seed oil and GLA are DNA safe (non-genotoxic) and antimutagenic compared to hydrogen peroxide, thereby confirming their antioxidant capacity; iii) borage seed oil and GLA exhibited cytotoxic activity in low doses (IC_50_ of 1 µl/ml and 0.087 mM, respectively) iv) Low doses of borage seed oil (0.19%) increased the health span of *D. melanogaster*; and v) GLA significantly decreased the life span of *D. melanogaster*.

Based on the antimutagenic and cytotoxic effects along with the ability to increase the health span, we propose the use of borage seed oil, rather than GLA, as a substance with antimutagenic/anticarcinogenic properties because it protects DNA by modulating the *in vivo* oxidative genetic damage in *D. melanogaster*, increases the health span and exerts *in vitro* cytotoxic activity towards promyelocytic HL60 cells.
